# The impact of perceived social support on athletic engagement among elite track and field athletes: an integrated perspective based on self-determination theory and social resources theory

**DOI:** 10.3389/fpsyg.2026.1763813

**Published:** 2026-01-23

**Authors:** Xue Li, Hengyu Li, Jin Hwang, Weijie Gao

**Affiliations:** 1 Department of Physical Education, Jeonbuk National University, Jeonju, Republic of Korea; 2 College of Physical Education, Northeast Electric Power University, Jilin, China

**Keywords:** athletic engagement, elite TF athletes, mediation, perceived social support, role identity

## Abstract

**Background:**

From the perspectives of Self-Determination Theory (SDT) and Social Resource Theory (SRT), social support is regarded as a crucial contextual factor influencing athletes’ psychological functioning and engagement. However, the mechanisms through which perceived social support affects athletic engagement, particularly the mediating role of role identity, remain insufficiently explored.

**Purpose:**

This study aimed to examine the relationship between perceived social support and athletic engagement among elite track and field (TF) athletes, and to investigate the mediating role of role identity from an integrated SDT and SRT framework.

**Results:**

The results indicated that: (1) perceived social support and its dimensions were significantly and positively associated with athletic engagement among elite TF athletes; (2) role identity was positively related to athletic engagement and partially mediated the relationship between perceived social support and athletic engagement; and (3) among the four dimensions of social support, esteem support and tangible support demonstrated the strongest and largely comparable total effects on athletic engagement, whereas emotional support and informational support showed relatively weaker associations.

**Conclusion:**

These findings suggest that perceived social support plays both direct and indirect roles in enhancing athletic engagement among elite track and field athletes, with role identity serving as a key mediating mechanism. Strengthening esteem and tangible support, as well as fostering athletes’ role identity, may be effective strategies for promoting sustained athletic engagement.

## 1 Introduction

In recent years, China has been actively advancing the integration of sports and education strategy. The policy document Opinions on Deepening the Integration of Sports and Education to Promote the Healthy Development of Young People (2020) explicitly emphasizes the need to “promote the coordinated development of students’ academic learning and physical exercise.” The construction of high-level university sports teams has become a key measure for implementing this national strategy (Shi et al., 2024). It is against this background that the effectiveness of deeper and more sustainable engagement of the elite athlete in the process of Athletic Engagement has become a usual line of interest to both scholars and practitioners. Athletic engagement as a concept is the result of the positive psychology which is a lasting and positive cognitive and emotional experience in the process of engaging in athletic activity (Yang, 2022). This is primarily characterized by confidence, dedication, vigor and enthusiasm (Lonsdale et al., 2007). When applied to sports, psychological resilience, motivation types, social support and coaching behaviors are several factors that affect the level of an individual being engaged in sports (Zhang et al., 2016; Li et al., 2020; Gao et al., 2021). Besides, athletic involvement is proven to be beneficial to the training state of athletes, their performance, and self-efficacy (Cui, 2022). Thus, to increase the result in athletic performance, there is a need to increase the level of athletic engagement of individuals. As the notion of the collaboration between sports and education is growing in China, school sports and competitive sports have contributed to the change of the model of nurturing elite sports reserve talents. Simultaneously, academic-conflicts are common among student-athletes, as they always have to alternate between being a student and an athlete, and this usually results in role conflicts and identity dilemmas (Yin et al., 2024). A relatively high level of role identity of people, the commitment to a role, their self-confirmation, is a valuable psychological resource of balance in academic and training activities, and athletic engagement promotion (Edison et al., 2021). Perceived social support is a concept that is used to describe the extent of perceived understanding, care, respect, and support of others or the social scenario by which an individual can identify an impact of perceived social support on the athletic engagement (Ye et al., 2016). The proposed study aims to determine the influence of the perceived social support on the athletic engagement and participation of elite track and field (hereinafter referred to as TF) athletes, and identify means of effectively increasing their engagement in the training and participation that can contribute to both theoretical and practical higher education of physical education and sport in Chinese higher universities.

### 1.1 Theoretical framework and research hypotheses

The present study draws on Self-Determination Theory (SDT) and Social Resource Theory (SRT) to develop an integrative framework for examining the associations between perceived social support, role identity, and athletic engagement among elite track and field athletes. Rather than proposing a definitive causal mechanism, this framework is intended to organize existing theoretical perspectives and empirical findings into a coherent conceptual model that can be statistically examined using cross-sectional data.

#### 1.1.1 Self-Determination Theory (SDT)

The Self-Determination Theory (SDT) was initially put forth by Ryan and Deci (2000). The perceived social support is one of the external environmental factors of SDT applied in sports (Xiang, 2013). SDT has been used in other areas including sports (Xiang, 2013), education (Hu and Zhu, 2025), healthcare (Zhang et al., 2022), and economics (Ma et al., 2018). When athletes feel understanding, trust, and support from their coaches, teammates, and families, their autonomy is respected. When they receive effective guidance and positive feedback, their competence is enhanced. When they become integrated into team relationships and experience recognition and emotional connection, their relatedness needs are satisfied (Coatsworth and Conroy, 2009; Jakobsen, 2023; Teixeira et al., 2012). A high level of athletic engagement is the external manifestation of the satisfaction of these three basic psychological needs (Ryan and Deci, 2000). At the same time, when elite TF athletes experience psychological satisfaction within a socially supportive environment, they are able to internalize external learning or training demands as part of their self-value, thereby reinforcing a salient ‘learner–athlete’ role identity. Role identity thus represents the psychological outcome of motivation internalization (Chu and Zhang, 2019). Therefore, from the perspective of SDT, prior literature suggests that perceived social support may be associated with psychological need satisfaction and the internalization of role identity, which in turn may relate to athletic engagement. However, this study does not directly test the full motivational sequence proposed by SDT.

#### 1.1.2 Social Resource Theory (SRT)

Social Resource Theory (SRT) explains the essential mechanism of social support from the perspective of social exchange. The theory suggests that social interaction is essentially a process of exchanging and utilizing social resources. Individuals can obtain six primary types of resources through different social relationships: love, status, information, services, goods, and money (Foa, 1971). Different forms of social resources satisfy individuals’ needs at different levels, and thus influence individuals’ psychological feelings and behavioral performance (Amorsen et al., 2025). In the sports context, SRT offers a social exchange perspective to explain perceived social support. Throughout their learning and training, elite athletes may gain different types of social resources from teachers, peers, coaches, and family members such as emotional resources (e.g., understanding and care), status resources (e.g., recognition and respect), and informational resources (e.g., understanding, feedback) (Rees et al., 2025). When individuals feel that they have a positive perception of the availability and value of these social resources, they will report a higher level of perceived social support. At the same time, the process of gaining and exchanging social resources will strengthen individuals’ social role identity (Stryker and Burke, 2000). Previous studies have indicated that, as elite athletes continue to receive support and encouragement from others and feedback from coaches and peers, they gradually recognize their value and significance in the two roles of “learner” and “athlete” and thus form a stable role identity (Bruner et al., 2021). This role identity can make them feel a greater sense of belonging and self-efficacy, and encourage sustained athletic engagement in training and competition.

**Figure 1:**
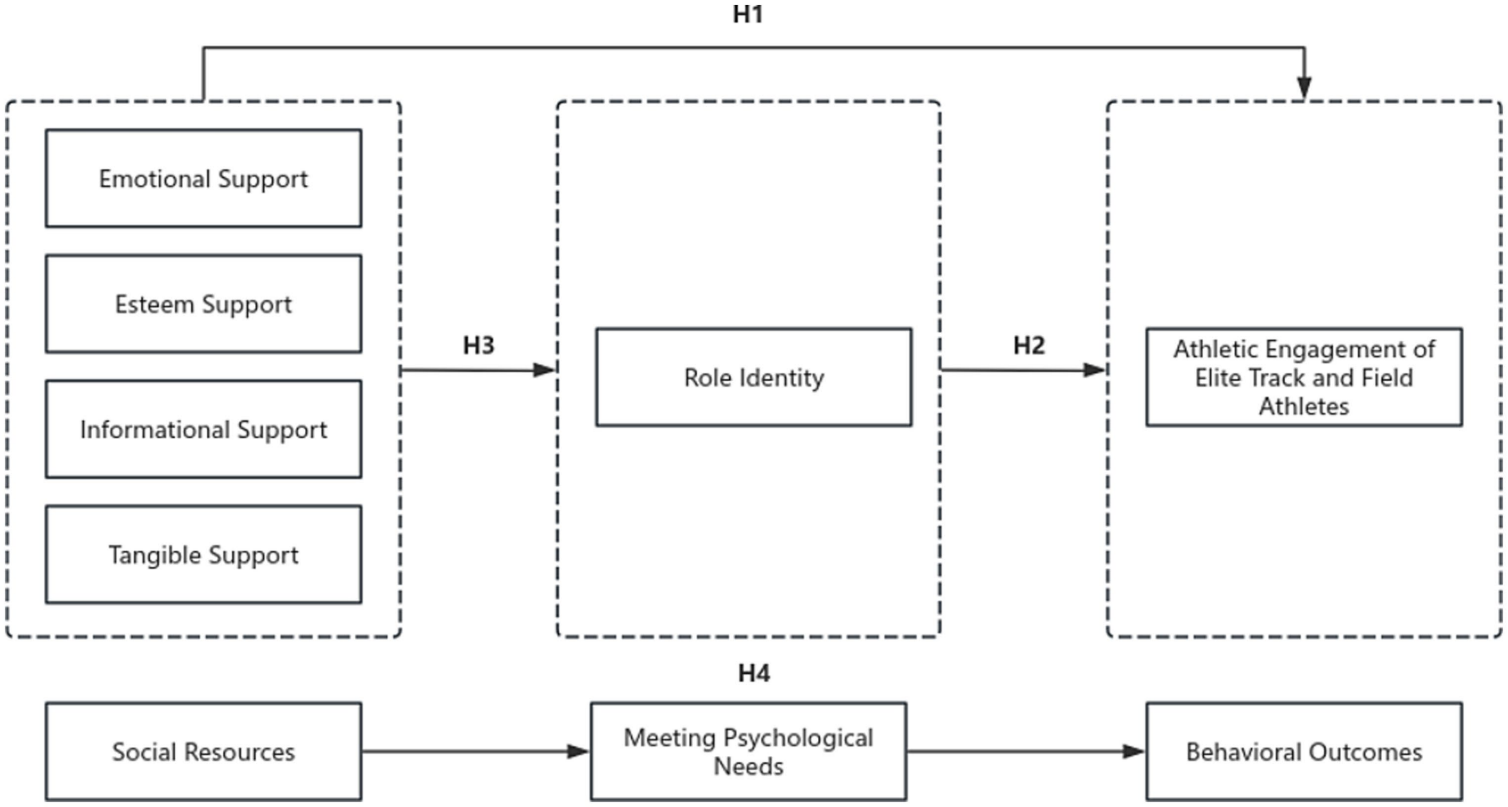
The integrated research model based on Self-Determination Theory (SDT) and Social Resource Theory (SRT).

Therefore, combining SDT and SRT can provide a more comprehensive explanation for how social support influences athletes’ engagement in training and competition. According to SRT, individuals gain multidimensional resources from their social networks, and the process of resource fulfillment is consistent with the psychological need satisfaction process emphasized by SDT. That is, the resources of relatedness and care are fulfilled by the informational resources and feedback provided by coaches and peers for athletes in terms of need for competence; the understanding provided by family and school fulfills the need for autonomy in terms of the support given by family and school to athletes; and the understanding and autonomy support provided by family and school fulfill the need for autonomy. When athletes perceive and internalize these resources, they may develop a stronger sense of identity with their roles, this is related to more advanced training and greater engagement to competitions (Williams et al., 2013). In summary, this study draws on SRT to conceptualize perceived social support as a set of multidimensional social resources, and on SDT to inform the interpretation of the psychological associations linking social support, role identity, and athletic engagement. Together, these perspectives are integrated into a conceptual framework that organizes existing findings and allows for the statistical examination of the associations among these constructs. As illustrated in Figure 1, the proposed model examines whether role identity statistically mediates the association between perceived social support and athletic engagement, thereby offering a theoretical reference for understanding academic–training balance and psychological adjustment among elite track and field athletes.

#### 1.1.3 The direct relationship between perceived social support and athletic engagement among elite TF athletes

Social support is the help that people get whether spiritual or material in form of their family, friends, and social organizations. It shows the intimacy and excellence of a person being in society and is broadly split into two categories, i.e., objective support and subjective support (Xiao, 1994). Perceived social support, as a part of subjective support, compares to the subjective attitude and judgment of support as provided by the coaches, teammates, family members, and peers. It is how much the external social resource is psychologically perceived and internalized by the athletes in the sports setting. Perceived social support can be discussed as the vital factor that facilitates positive psychological states of athletes and sport performance. Through the Self-Determination Theory (SDT), intrinsic motivation can be increased when athletes perceive that their most fundamental psychological needs are met, namely, their needs are autonomy, competence, and relatedness, with which one will find increased engagement in athletics, enthusiasm, concentration, and continued engagement. Indicatively, the findings of Shi et al. (2025) showed that social support is a predictive factor of athletic involvement among young athletes. Likewise, Suguis and Elic (2025) affirmed that social support is a significant foreteller of training engagement in Filipino collegiate athletes who exhibit positive effects across all dimensions of engagement subdimensions. As discussed above, there is a close relationship between social support and athletic engagement. Most existing studies have focused on the impact of social support on the engagement of professional athletes, while relatively few have examined how perceived social support influences athletic engagement among elite TF Athletes. This research gap provides an opportunity for further investigation in the present study. Based on the above theoretical discussion, the following hypothesis is proposed: H1 (a–d): Perceived social support—including emotional support, esteem support, informational support, and tangible support—has a positive effect on the athletic engagement of elite TF Athletes.

#### 1.1.4 The mediating role of role identity

Role identity describes the individual meaning of belonging and psychological sense to the society. Role identity in the case of elite TF athletes shows the way people see themselves, perceive their role, and value assessment of the dual roles of a student and an athlete in the particular social context or environment. Studies have proposed the role identity as being the weight of the athletic role, the thought, and involvement of the academic role. The relative salience of the two roles reflects the subjective importance individuals attach to their academic and athletic identities in the context of academic–athletic demands. Which may reinforce athletes’ overall role identity strength and be associated with higher levels of intrinsic motivation and engagement. SDT demonstrates that when the social environment helps fulfill individuals with basic psychological needs of autonomy, competence, and relatedness, the externality support is transferred to self-identity and is associated with intrinsic motivation to positive behaviors. Reifsteck et al. (2013) declared that role identity was a significant prediction of the degree of participation in activity based on a study of former elite athletes. In a meta-analysis of Chinese adolescent athletes, it was evident that role identity is a significant predictor of athletic engagement. Moreover, it was also identified that the more intense the role identity, the greater the intensity of focus and the engagement behaviors, and team performance in training, competitions, and team settings (Xie, 2021). These results also justify the notion that role identity represents an important psychological process that can be used to improve the athletic engagement of elite athletes. On this basis, the hypothesis below can be formulated: H2: Role identity is positively associated with athletic engagement.

Role identity has also been identified to be closely connected to perceived social support. In one of the studies carried out on college students, Lou (2010) indicated that the factors that drive role identity are both the internal factors (self-esteem) and the external environmental factors (sociocultural support, family support and support of friends), which harmoniously lead to the development of role identity. On the same note, Xiao et al. (2022) stipulated that external social environmental factors have a significant impact on the intensity of role identity in the students. Within the sports setting, Bruner et al. (2021) identified that the greater amount of perceived social support the more prominent the role identity of youth players was. Based on the existing literature, the following hypothesis is proposed: H3: Perceived social support—including emotional support, esteem support, informational support, and tangible support—has a positive effect on role identity.

According to the above research, perceived social support is correlated with role identity and athletic engagement According to SDT and SRT, social support, as an external resource input, satisfies athletes ‘psychological needs through emotional and information exchange; athletes form and strengthen role identity in this process, thus stimulating intrinsic motivation and self-efficacy, and finally show higher level of athletic engagement. Therefore, the following hypothesis is proposed: H4: Role identity is expected to play a mediating role in the association between perceived social support (emotional support, esteem support, informational support, and tangible support) and athletic engagement.

According to the SDT and the SRT, the research model was created in this study (Figure 1) where perceived social support is the antecedent variable, role identity is the mediating variable, and athletic engagement is an outcome variable to explore the relationship between perceived social support and athletic engagement in elite TF Athletes.

## 2 Research subjects and tools

### 2.1 Research subjects

College track and field athletes from universities in Heilongjiang, Jilin, and Liaoning provinces were recruited for this study. A total of 400 questionnaires were distributed, of which 381 were returned (response rate = 95.25%). After data screening, 339 questionnaires were retained as valid, yielding an effective response rate of 89%. This research was supported by the IRB (Approval No. KIRD-2024-03-31-J-E-183) of Jeonbuk National University, Republic of Korea, and the ethical standards of 1964 Helsinki Declaration and its later amendments or revisions. Informed consent was obtained from all participants.

### 2.2 Research tools

#### 2.2.1 Perceived social support scale

Perceived social support was measured using the Perceived Available Support in Sport Questionnaire (PASS-Q) developed by Freeman et al. (2011). The questionnaire consists of 16 items rated on a 5-point Likert scale and includes four dimensions: emotional support, esteem support, informational support, and tangible support. The total score is the sum of all dimension scores, with higher scores indicating a greater level of perceived available social support. In this study, the Cronbach’s *α* coefficient of the scale was 0.865, indicating good internal consistency reliability. The structural validity of the scale was also satisfactory, with the following fit indices: *χ*^2^/df = 1.693, GFI = 0.941, AGFI = 0.918, RMSEA = 0.045, CFI = 0.981, NFI = 0.955, and IFI = 0.981, demonstrating that the scale had good construct validity.

#### 2.2.2 Role identity scale

The measure of role identity was created with the help of the Academic and Athletic Identity Scale (AAIS) created by Yukhymenko-Lescroart (2014). The AAIS is a questionnaire of 11 questions in two subscales- academic identity and athletic identity and a 6-point Likert scale. The highest score is the sum of the two subscale scores and the greater the score the greater the degree of role identity. In the present study, the total AAIS score was used as an indicator of overall role identity strength, reflecting the salience of academic and athletic identities, rather than the degree of role integration, identity consistency, or role commitment. In the current study the Cronbach’s *α* coefficient of the scale was 0.836, good internal consistency reliability. The scale had also satisfactory structural validity and had the following fit indices, *χ*^2^/df = 1.599, GFI = 0.963, AGFI = 0.944, RMSEA = 0.042, CFI = 0.985, NFI = 0.961 and IFI = 0.985, which show that the scale was of good construct validity.

#### 2.2.3 Athletic engagement scale

Athletic engagement was assessed through Athlete Engagement Questionnaire (AEQ) developed by Lonsdale et al. (2007). The scale is composed of 16 items in four dimensions: confidence, dedication, vigor, enthusiasm. All items were scored on a 5-point Likert scale from 1 (strongly disagree) to 5 (strongly agree) and higher scores indicated a higher level of athletic engagement. In the present study, Cronbach’s α coefficient of the scale was calculated as 0.859 for good internal consistency reliability. In addition, the structural validity of the scale is acceptable with the following fit indices: *χ*^2^/df = 1.610, GFI = 0.947, AGFI = 0.925, RMSEA = 0.042, CFI = 0.975, NFI = 0.937, IFI = 0.975 and good construct validity.

### 2.3 Analysis process

Analysis of data was done in the SPSS and AMOS software. The analysis was done using descriptive statistics to begin with to know the basic features of the sample. Then, the mediating model was tested with the help of regression analysis and the relationships between perceived social support, role identity and athletic engagement were observed. The Bootstrap method was utilized to ascertain the importance of the mediating effect to be sure that the results are robust.

## 3 Results analysis

### 3.1 Demographic variables statistics

The number of participants involved in this study was 339 and a breakdown in Table 1 indicates that there were 173 males (51%), and 166 females (49%). In terms of years of training, 86 athletes (25.4%) had been training between 3 and 5 years, 179 athletes (52.8%) between 6 and 8 years, and 74 athletes (21.8%) took training over 9 years. Regarding athletic level, 85 (25.1%) participants were at the National Level 1 and 254 (74.9%) participants were at the National Level 2. Overall, the sample included elite TF Athletes of different genders, training years, and athletic levels.

**Table 1:** Demographic information of the sample.

Category	Option	Frequency	Percentage (%)
Gender	Male	173	51
	Female	166	49
Years of training	3–5 years	86	25.4
	6–8 years	179	52.8
	More than 9 years	74	21.8
Athletic level	National Level 1	85	25.1
	National Level 2	254	74.9
Total		339	100

### 3.2 Common method bias test

This study employed Harman’s one-factor test to examine common method bias. All variable measurement items underwent factor analysis together, revealing five factors with eigenvalues exceeding 1. The largest factor explained 28.974% of the variance, falling below the standard critical threshold of 40% (Tang and Wen, 2020). However, it is necessary to point out that the Harman single-factor test alone can only provide a rough judgment and cannot fully rule out the influence of common method bias. Therefore, the results of this study cannot be completely understood as being unaffected by common method bias; it should be regarded as that under the current conditions, no dominant common method bias test has been observed.

### 3.3 Descriptive statistics and correlation analysis

As shown in Table 2, perceived social support was positively correlated with emotional support, esteem support, informational support, tangible support, role identity, and athletic engagement. Emotional support was positively correlated with esteem support, informational support, tangible support, role identity, and athletic engagement. Esteem support showed positive correlations with informational support, tangible support, role identity, and athletic engagement. Informational support was positively correlated with tangible support, role identity, and athletic engagement. Tangible support was positively correlated with role identity and athletic engagement. Finally, role identity was positively correlated with athletic engagement. These results provide preliminary support for the study.

**Table 2:** Means, standard deviations, and correlation coefficients between variables (*N* = 339).

Variable	*M*	SD	1	2	3	4	5	6	7
1	63.274	10.622	1						
2	15.950	3.121	0.879**	1					
3	15.779	2.955	0.827**	0.753**	1				
4	15.723	2.778	0.799**	0.673**	0.798**	1			
5	15.844	2.851	0.824**	0.729**	0.822**	0.819**	1		
6	50.248	9.315	0.376**	0.312**	0.375**	0.449**	0.366**	1	
7	62.068	9.633	0.396**	0.414**	0.311**	0.457**	0.496**	0.349**	1

1 = Perceived Social Support, 2 = Emotional Support, 3 = Esteem Support, 4 = Informational Support, 5 = Tangible Support, 6 = Role Identity, 7 = Athletic Engagement. **p < 0.01.

### 3.4 The effect of perceived social support on athletic engagement among elite TF athletes

Following the mediation testing procedure recommended by Wen et al. (2004), regression analysis treated perceived social support as the independent variable and Athletic Engagement as the dependent variable. Findings revealed that among elite TF Athletes, perceived social support was significantly and positively associated with athletic engagement (*β* = 0.296, *t* = 5.694, *p* < 0.001), indicating that perceived social support was significantly and positively associated with athletic engagement. This study further examined whether the four dimensions of perceived social support—emotional support, esteem support, informational support, and tangible support—independently influenced elite TF Athletes’ athletic engagement and role identification. Controlling for variables such as gender, years of training, and athletic level, a total of 11 regression analyses were conducted:

(1) When athletic engagement was used as the dependent variable:

In Model 1, gender, years of training, and athletic level had no significant effect on athletic engagement among elite TF athletes (*F* = 0.802, *p* > 0.05).

In Model 2, emotional support was significantly associated with athletic engagement (*β* = 0.216, *p* < 0.001), thereby supporting Hypothesis H1a.

In Model 3, esteem support was significantly related to athletic engagement among elite TF athletes (*β* = 0.319, *p* < 0.001), thereby supporting Hypothesis H1b.

In Model 4, informational support was significantly and positively associated with athletic engagement among elite TF athletes (*β* = 0.262, *p* < 0.001), thereby supporting Hypothesis H1c.

In Model 5, tangible support showed the strongest association with athletic engagement (*β* = 0.310, *p* < 0.001), thus supporting Hypothesis H1d.

In Model 6, role identity was significantly related to athletic engagement (*β* = 0.358, *p* < 0.001), thereby supporting Hypothesis H2.

(2) When role identity was used as the dependent variable:

In Model 7, gender (*β* = 0.139, *p* < 0.05) and years of training (*β* = 0.111, *p* < 0.05) had significant positive effects on role identity among elite TF athletes, indicating that male athletes and those with longer training experience showed higher levels of role identity.

In Model 8, emotional support had a significant positive effect on role identity (*β* = 0.209, *p* < 0.001), thereby supporting Hypothesis H3a.

In Model 9, esteem support had a significant positive effect on role identity (*β* = 0.273, *p* < 0.001), thus supporting Hypothesis H3b.

In Model 10, informational support had a significant positive effect on role identity (*β* = 0.235, *p* < 0.001), thereby supporting Hypothesis H3c.

In Model 11, tangible support had a significant positive effect on role identity (*β* = 0.241, *p* < 0.001), thus supporting Hypothesis H3d (Table 3).

**Table 3:** Test of direct effects.

Variable	Athletic engagement						Role identity				
	Model 1	Model 2	Model 3	Model 4	Model 5	Model 6	Model 7	Model 8	Model 9	Model 10	Model 11
Gender	−0.023	−0.056	−0.065	−0.057	−0.074	−0.070	0.139*	0.107*	0.103	0.089	0.108*
Years of training	−0.016	−0.003	0.006	−0.008	0.022	0.017	0.111*	0.123*	0.130*	−0.017	−0.117*
Athletic level	−0.078	0.048	0.07	0.069	−0.320	−0.025	0.014	−0.015	0.007	0.019	0.006
Emotional support		0.216***						0.209***			
Esteem support			0.319***						0.273***		
Informational support				0.262***						0.235***	
Tangible support					0.310***						0.241***
Role identity						0.358***					
*R* ^2^	0.007	0.052	0.107	0.074	0.099	0.135	0.026	0.058	0.098	0.07	0.083
Δ*R*^2^	−0.002	0.04	0.096	0.063	0.088	0.125	0.017	0.056	0.088	0.059	0.072
*F*	0.802	4.547**	9.964***	6.719***	9.155***	13.067***	2.974*	6.049***	9.109***	6.285***	7.528***

All coefficients reported are standardized regression coefficients (β). *p < 0.05, **p < 0.01, ***p < 0.001.

### 3.5 Testing for mediation effects

**Figure 2:**
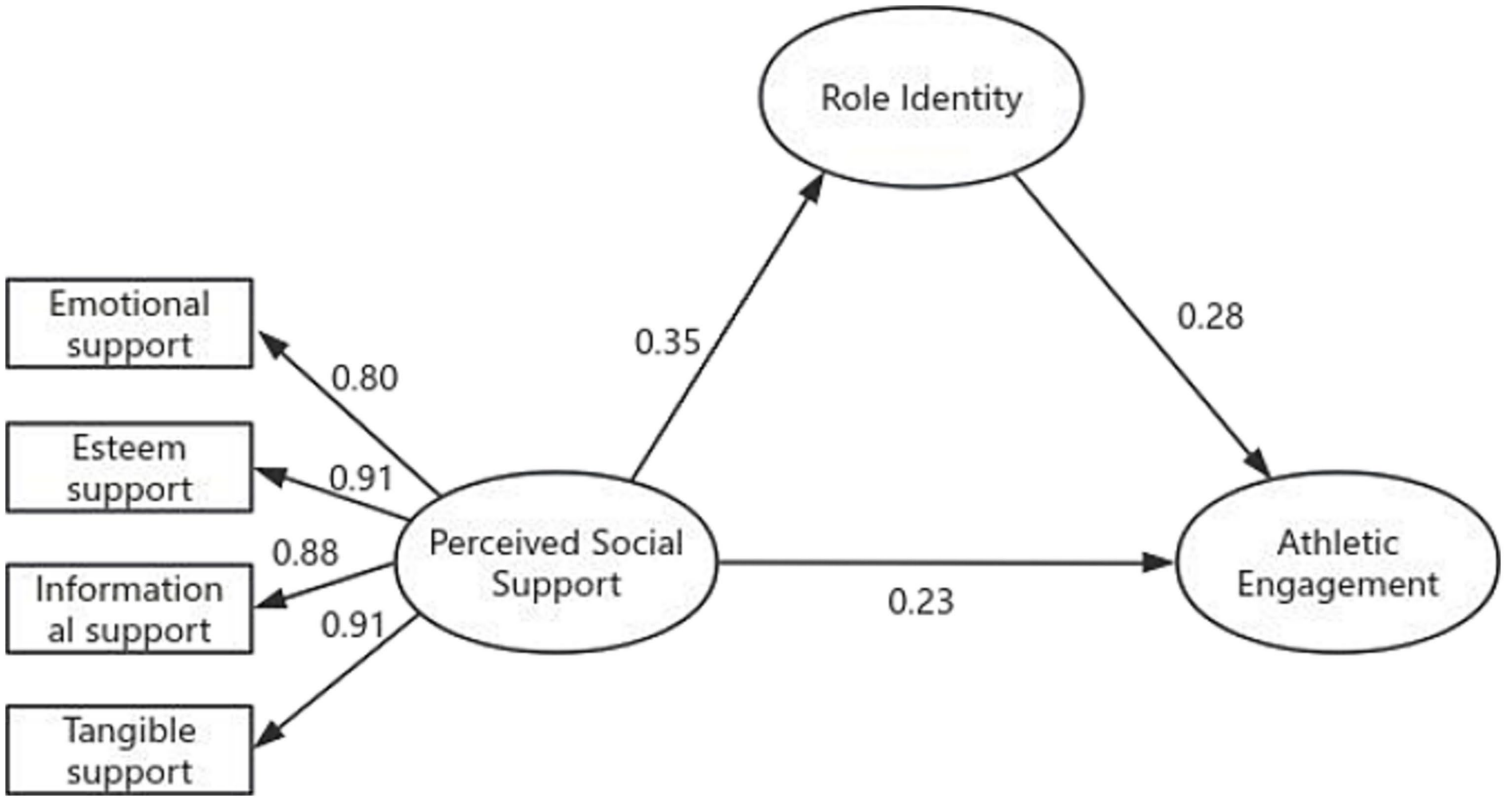
Structural equation model illustrating the associations among perceived social support, role identity, and athletic engagement. Standardized path coefficients are shown.

The structural equation modeling of perceived social support, role identity and athletic engagement was conducted using AMOS 29.0 software. The analysis revealed that the fitting indices were quite satisfactory: *x*^2^/df = 1.379, GFI = 0.975, AGFI = 0.957, RMSEA = 0.034, CFI = 0.995. As shown in Figure 2, perceived social support was positively associated with role identity (*β* = 0.35, *p* < 0.001) and athletic engagement (*β* = 0.23, *p* < 0.001). Role identity was also positively associated with athletic engagement (*β* = 0.28, *p* < 0.001). These results indicate that role identity partially mediated the association between perceived social support and athletic engagement.

This SEM analysis provides a holistic test of the proposed theoretical framework and complements the regression-based mediation analyses by simultaneously estimating the structural paths among the latent constructs.

The four dimensions of perceived social support – emotional support, esteem support, informational support, and tangible support – are four different types of social support. Current research mainly focuses on the overall relationship between perceived social support and athletic engagement, while studies on the relationships between different types of perceived social support and athletic engagement are relatively insufficient (Tao and Yu, 2025). Therefore, based on this, this study further explores the impact of different types of perceived social support on athletic engagement.

Taking the various dimensions of perceived social support as independent variables, role identity as the mediating variable, and athletic engagement as the dependent variable, the data were modeled and analyzed. Currently, there are two main methods for testing mediation effects involving multiple independent variables. The first approach is to include all independent variables in a single model; however, this method carries the risk that the predictive effects of different variables may offset each other. The second approach is to build separate models for each independent variable and analyze them individually. This latter method is more commonly used in current research. In this study, the second approach was adopted, and the Bootstrap method was applied using the SPSS macro program developed by Preacher and Hayes to test the mediating effect of role identity.

As shown in Table 4, the total, direct, and indirect effects of emotional support on athletic engagement were 0.662, 0.454, and 0.208, respectively. The direct effect accounted for 68.58% of the total effect, and the indirect effect accounted for 31.42%. The 95% confidence intervals (0.339, 0.985), (0.140, 0.767), and (0.084, 0.368) did not include zero, indicating that role identity partially mediated the relationship between emotional support and athletic engagement among elite TF Athletes. Thus, Hypothesis H4a was supported. For esteem support, the total, direct, and indirect effects were 1.015, 0.760, and 0.078, respectively. The direct effect accounted for 74.88% of the total effect, and the indirect effect accounted for 7.72%. The 95% confidence intervals (0.683, 1.347), (0.429, 1.091), and (0.036, 0.128) did not include zero, suggesting that role identity partially mediated the relationship between esteem support and athletic engagement. Therefore, Hypothesis H4b was supported. For informational support, the total, direct, and indirect effects were 0.891, 0.629, and 0.262, respectively. The direct effect accounted for 70.58% of the total effect, and the indirect effect accounted for 29.42%. The 95% confidence intervals (0.275, 0.983), (0.108, 0.437), and (0.654, 1.346) did not include zero, indicating that role identity partially mediated the relationship between informational support and athletic engagement. Thus, Hypothesis H4c was supported. For tangible support, the total, direct, and indirect effects were 1.000, 0.738, and 0.262, respectively. The direct effect accounted for 73.82% of the total effect, and the indirect effect accounted for 26.18%. The 95% confidence intervals (0.654, 1.346), (0.395, 1.081), and (0.118, 0.430) did not include zero, confirming that role identity partially mediated the relationship between tangible support and athletic engagement. Therefore, Hypothesis H4d was supported.

**Table 4:** The effects of emotional support, esteem support, informational support, and tangible support on athletic engagement.

Variable	Effect type	Effect	SE	Bias-corrected 95% CI	
				Lower	Upper
Emotional support	Total effect	0.662	0.164	0.339	0.985
	Direct effect	0.454	0.160	0.140	0.767
	Indirect effect	0.208	0.073	0.084	0.368
Esteem support	Total effect	1.015	0.169	0.683	1.347
	Direct effect	0.760	0.168	0.429	1.091
	Indirect effect	0.078	0.024	0.036	0.128
Informational support	Total effect	0.891	0.183	0.532	1.250
	Direct effect	0.629	0.180	0.275	0.983
	Indirect effect	0.262	0.084	0.108	0.437
Tangible support	Total effect	1.000	0.176	0.654	1.346
	Direct effect	0.738	0.175	0.395	1.081
	Indirect effect	0.262	0.079	0.118	0.430

## 4 Discussion

### 4.1 The direct effect of perceived social support on athletic engagement among elite TF athletes

The findings of the present study revealed that the perceived social support and its dimensions had significant and positive predictors of athletic engagement in elite TF Athletes and the higher the perceived social support, the higher the athletic engagement. This finding aligns with the findings of Xiao and Jiang (2025). From the perspective of SDT, perceived social support meets the key external environmental conditions for the three basic psychological needs (autonomy, competence, and relatedness) of elite TF athletes. For elite TF athletes who have been in a state of “academic–training conflict” for a long time, the significance of social support far exceeds ordinary interpersonal care. When athletes perceive understanding and support from coaches, teammates, and the school level, their sense of autonomy that has been squeezed under dual pressures will be effectively restored. When athletes’ progress and efforts receive positive affirmation from coaches and teammates, their sense of competence will be significantly enhanced. And the unconditional support and encouragement from families and teams provide an indispensable sense of relatedness for them in the high-pressure and highly competitive environment. According to the core viewpoints of SDT, the satisfaction of these three basic psychological needs is the prerequisite for high-quality intrinsic motivation, which can directly translate into the core dimensions of sports engagement: enthusiasm for training, vitality experienced in competitions, dedication to the sports career, and confidence in achieving goals (Liu et al., 2025). From the perspective of SRT, athletic engagement is closely linked to social resources (Mira et al., 2023). Perceived social support is not only a subjective perception but also an accessible form of resource, including emotional, informational, and tangible support. In this study, perceived social support is classified as a type of social resource (Ma et al., 2025). For elite athletes, where they must cope with academic demands and examination stress while also maintaining training loads, competition performance, and athletic development. When external social resources are insufficient, individuals’ internal resource reserves may quickly deplete, leading to psychological exhaustion and a decline in engagement (Hobfoll et al., 2018). Conversely, when elite athletes perceive adequate social support—such as understanding from family, guidance from coaches, encouragement from teammates, and institutional support from schools—these relational resources may function as positive psychological resources that are associated with better psychological experiences and higher levels of athletic engagement even when facing academic–training conflict (Berg and Warner, 2019). In conclusion, the direct impact of perceived social support on athletic engagement has a dual internal logic. It can provide motivation for athletic engagement through the “psychological mechanism” path, which satisfies basic psychological needs; it can also provide behavioral ability for athletic engagement through the “functional mechanism” path, which buffers personal resources. For high-level track and field athletes who are in a special stress situation of “academic–training conflict,” the synergy of these two paths constitutes the fundamental reason why they can maintain their athletic engagement in a high-pressure environment.

### 4.2 The mediating role of role identity

The results suggested a partial mediation pattern, with role identity statistically accounting for part of the association between perceived social support and athletic engagement. On the one side, the use of social support may be directly associated with athletic engagement; in addition, it may also be related to higher levels of engagement through its association with role identity, which in turn may be linked to athletes’ psychological experiences and behavioral tendencies (Haslam et al., 2008). Regarding the Social Identity Theory (SIT), the responses of the athletes to social support act as an indicator of identity-strengthening, which enhances a positive engagement in training and competition due to the connection to the importance and social meaning of the participants in the roles. Meanwhile, among the suggestions of the SRT, the social support, as one of the essential relational resources, is a critical external factor that contributes to the ability of the athletes to deal with the stress associated with academic-training conflict. Elite TF athletes who report higher levels of perceived social support also tend to report greater psychological resources, which are associated with higher levels of engagement in both training and academic contexts. Within this context, social resources and role identity appear to be closely associated with athletes’ psychological experiences, which may be related to differences in engagement across academic and athletic domains. Under the background of the continuous deepening of the development strategy of integration of sports and education in China, the dual role of social resources and role identification is particularly important. As one of the core groups in the implementation of the policy of integration of sports and education, high-level athletes not only need to achieve results in sports competitions, but also emphasize their educational development and personality molding. A high level of perceived social support environment can help athletes strengthen their role identity as “student-athletes” and reduce the pressure of learning and training conflicts, thus promoting higher-quality athletic engagement in both academic and training contexts. It should be noted that role identity in the present study was operationalized as the overall strength of academic and athletic identities, as measured by the summed score of the AAIS, rather than as an index of role integration, identity consistency, or role commitment. Accordingly, the observed mediation effect should be interpreted as reflecting the salience of dual role identities, rather than a psychological process of identity integration or conflict resolution.

### 4.3 A comparison of the effects of different types of perceived social support on athletic engagement among elite TF athletes

Results of this study indicated that different forms of perceived social support, such as emotional support, esteem support, informational support, and tangible support, all significantly influenced athletic engagement of elite TF athletes. However, different types of perceived social support had different total effects on athletes’ engagement. Based on the total effects estimated in the mediation analyses, esteem support and tangible support exhibited the strongest and largely comparable associations with athletic engagement, whereas emotional and informational support showed relatively weaker total associations. This difference is not simply a ranking of the effect sizes; rather, it reflects that different types of social support exert their influence on elite TF athletes through distinct psychological mechanisms. Firstly, the reason why esteem support have a significant impact on athletic engagement in sports is that they meet the athletes’ needs for “autonomy” and “competence.” These two needs are precisely the core demands of SDT “esteem” is not only interpersonal care but also the recognition of the athletes’ professional abilities, identity values, and the efforts they have made. The esteem from teammates and coaches directly serves as a powerful catalyst for internal motivation. Secondly, tangible support is almost equivalent to esteem support, which reveals the crucial role of the Social Resource Theory (SRT) in the “academic–training conflict” situation of high-level athletes. Tangible support (such as training equipment, medical care and rehabilitation, etc.) constitutes the strategic resources necessary for high-level athletes to maintain their dual identities. In a high-resource-consuming sports environment, tangible support is not merely a “material provision” but rather a “resource consumption buffering mechanism.” It effectively reduces the psychological pressure on athletes caused by insufficient resources by eliminating external environmental obstacles, allowing them to devote more time and energy to the training itself, thereby directly enhancing the sustainability of their athletic engagement. Finally, emotional support and informational support place more emphasis on the “basic enabling conditions.” Generally, emotional support refers to the support and assistance that individuals receive from coaches, families, and teammates. When athletes experience the understanding of their coaches, the care of their teammates, and the trust of their families during training and competitions, they will have positive emotional responses, thereby strengthening their psychological sense of relatedness. This sense of belonging and acceptance enables athletes to psychologically identify more with the role of “student-athlete” and be willing to continuously invest under the dual pressure of studies and training. Information support mainly helps athletes form clearer role cognition and goal positioning by providing guidance, feedback, and communication. Therefore, the combined effect of these two aspects contributes to the role identity of high-level athletes, thereby indirectly increasing their athletic engagement. In conclusion, the core contribution of this study lies in revealing the “dual-path model” of the impact of social support on the athletic engagement of high-level athletes. This model consists of the “direct driving” path, which is composed of esteem support and tangible support, and the “indirect empowering” path, which is composed of emotional support and informational support. In summary, through the collaborative effect of these two paths, the external efficacy of social support can be transformed into an internal motivation for enhancing sports engagement, thereby truly implementing the strategy of integrating sports and education.

## 5 Conclusion and implications

### 5.1 Conclusion

Based on the above research, The following conclusions are concluded: (1) demographic factors (including gender, athletic level, and years of training). There is no significant effect on the level of athletic engagement among elite track and field athletes. (2) Perceived social support was significantly associated with athletic engagement among elite track and field athletes. Among the four dimensions, esteem support and tangible support demonstrated the strongest and largely comparable associations with athletic engagement, whereas emotional and informational support exhibited relatively weaker associations. (3) Role identity had a positive effect on athletic engagement and served as a partial mediator between emotional support, esteem support, informational support, tangible support, and athletic engagement among elite TF athletes. It should be emphasized that, given the cross-sectional design and self-report nature of the data, the proposed pathways discussed above should be interpreted as patterns of association rather than as confirmed psychological processes.

### 5.2 Implications

Research implications: (1) perceived social support is strongly associated with athletic engagement among elite TF athletes. Therefore, universities, coaches, teammates, and family members should provide sufficient support and assistance to athletes, enabling them to engage in training and competition with greater enthusiasm and dedication. (2) Role identity is an important factor influencing the relationship between perceived social support and athletic engagement. Role identity not only directly enhances athletes’ engagement but also strengthens their identification with the “student-athlete” role when they receive support from their schools, coaches, teammates, and families. In turn, further promotes sustained athletic engagements. Thus, role identity serves as both a direct driver of engagement and a partial mediator between perceived social support and athletic engagement. (3) The results of this study indicated that different dimensions of perceived social support were differentially associated with athletic engagement, with esteem support and tangible support showing the strongest and largely comparable total associations, whereas informational and emotional support exhibited relatively weaker associations. Consistent with the findings reported in the Results and Discussion sections, esteem support and tangible support demonstrated the strongest and largely comparable total associations with athletic engagement. Therefore, in the context of university sports management and athletic training, particular emphasis should be placed on strengthening esteem support and tangible support. On the one hand, coaches and teachers should enhance athletes’ sense of self-worth through respectful communication, positive feedback, and recognition of achievement. On the other hand, universities should improve training and academic conditions, increase resource allocation efficiency, and ensure adequate time, facilities, and equipment for athletes. The combined presence of psychological encouragement and tangible support may represent an important practical avenue for promoting high-quality athletic engagement and the holistic development of elite track and field athletes.

## 6 Research limitations

In an integrated approach of Self-Determination Theory (SDT) and Social Resource Theory (SRT), this study explored the interconnection between perceived social support, role identity and athletic engagement among elite TF athletes and identified the partially mediation role of role identity. Nevertheless, various limitations are to be mentioned, which give recommendations on future research. To begin with, despite the popularity of SDT and SRT in the psychology discipline, there is a dearth of literature presenting the two theories together to uncover the mechanisms that affect an athlete to engage. The theoretical integration in the study is still at the initial level and has not yet given a full coverage of the possible mechanisms of interaction of the two theories. From a methodological perspective, although the present study employed structural equation modeling (SEM) to test the overall theoretical framework, dimension-specific mediation effects were further examined using regression-based bootstrap analyses. While this approach allowed for a more detailed comparison across different types of perceived social support, future research may extend the current model by estimating more complex SEM that simultaneously incorporate multiple support dimensions as parallel predictors. Such extensions may enable a more comprehensive comparison of effect sizes within a single structural framework. Second, as a result of time and geographical constraints, the sample and coverage was small mostly consisting of universities in Heilongjiang, Jilin and Liaoning Provinces, and there was representation scarcity of southern and central-western controls, and other sports disciplines. This might reduce the external validity of the results. In the future studies, larger and more heterogeneous sample with athletes of other geographical areas and other kinds of athletes (e.g., ball games) should be used to improve the external validity of the results. Thirdly, this study adopts a cross-sectional and self-report data collection method. Although preliminary tests for common method bias were conducted in the analysis, there may still be unavoidable influences. Therefore, the relationships between the variables reported in this study should be understood as the results presented in a specific sample and under a specific context. Future research can conduct further verification of the proposed paths in this study through longitudinal tracking, experimental research, etc. Lastly, in this study, role identity was assessed as the combined strength of academic and athletic identities, which did not allow distinction between role integration, identity conflict, or role commitment. Future research should adopt multidimensional or profile based approaches to more accurately capture the structural relationships between multiple identities.

## Data Availability

The raw data supporting the conclusions of this article will be made available by the authors, without undue reservation.
